# Supplementary material for: Association of Barriers, Fear of Falling and Fatigue with Objectively Measured Physical Activity and Sedentary Behavior in Chronic Stroke
Source: J Clin Med. 2021 Mar 23;10(6):1320. doi: 10.3390/jcm10061320 (PMC8005010; doi:10.3390/jcm10061320)
Supplement: Supplementary file 1 [file jcm-10-01320-s001.zip › Suplementary material 1.docx]

**Table S6.** Questionnaire to assess barriers to physical activity after stroke (BAPAS) (Drigny, et al., 2019).

| **Please fill in all fields** | **Strongly Disagree** | **Disagree** | **Slightly Disagree** | **Slightly Agree** | **Agree** | **Strongly Agree** |
| --- | --- | --- | --- | --- | --- | --- |
| 1. I lack information from the healthcare professional | 0 | 1 | 2 | 3 | 4 | 5 |
| 1. I have problems with transport | 0 | 1 | 2 | 3 | 4 | 5 |
| 1. I lack motivation | 0 | 1 | 2 | 3 | 4 | 5 |
| 1. I have a loss of muscle strength, paralysis | 0 | 1 | 2 | 3 | 4 | 5 |
| 1. I am afraid of having another stroke | 0 | 1 | 2 | 3 | 4 | 5 |
| 1. I have spasticity, muscle stiffness | 0 | 1 | 2 | 3 | 4 | 5 |
| 1. I am too exhausted | 0 | 1 | 2 | 3 | 4 | 5 |
| 1. I am afraid of falling | 0 | 1 | 2 | 3 | 4 | 5 |
| 1. I am not sporty | 0 | 1 | 2 | 3 | 4 | 5 |
| 1. I have other medical conditions | 0 | 1 | 2 | 3 | 4 | 5 |
| 1. I am feeling down | 0 | 1 | 2 | 3 | 4 | 5 |
| 1. I am slower | 0 | 1 | 2 | 3 | 4 | 5 |
| 1. I lack financial resources | 0 | 1 | 2 | 3 | 4 | 5 |
| 1. I am in pain | 0 | 1 | 2 | 3 | 4 | 5 |

Paired items belong to the physical barriers subscale (locomotor problems: items no. 2,4,6,8 and 12, and comorbidities: items no. 10 and 14). Odd items belong to the behavioural barriers subscale (fatigue and mood: items no. 5, 7 and 11, and motivation: items no.1, 3, 9 and 13)

Reference: Drigny, J.; Joussain, C.; Gremeaux, V.; Morello, R.; Van Truc, P.H.; Stapley, P.; Touzé, E.; Ruet, A. Development and validation of a questionnaire to assess barriers to physical activity after stroke: The Barriers to Physical Activity After Stroke Scale. *Arch. Phys. Med. Rehabil*. **2019**, 100, 1672–1679, doi:10.1016/j.apmr.2018.12.034.
