# Supplementary material for: Association of Barriers, Fear of Falling and Fatigue with Objectively Measured Physical Activity and Sedentary Behavior in Chronic Stroke
Source: J Clin Med. 2021 Mar 23;10(6):1320. doi: 10.3390/jcm10061320 (PMC8005010; doi:10.3390/jcm10061320)
Supplement: Supplementary file 1 [file jcm-10-01320-s001.zip › Suplementary material 2- proof.docx]

**Table S7.** Relationship between the physical barriers items from the Barriers to Physical Activity after Stroke Scale and Physical Activity and Sedentary behavior (n=57).

|  | **Sedentary time (%)** | | **Prolonged sedentary**  **time (%)** | | **LPA (%)** | | **MVPA (%)** | |
| --- | --- | --- | --- | --- | --- | --- | --- | --- |
|  | **ρ** | **p-value** | **ρ** | **p-value** | **ρ** | **p-value** | **ρ** | **p-value** |
| Locomotor problems | | | | | | | | |
| I have problems with transport | -0.020 | 0.882 | **0.348** | **0.008** | 0.101 | 0.454 | **-0.339** | **0.010** |
| I have a loss of muscle strength, paralysis | 0.037 | 0.783 | **0.414** | **0.001** | 0.080 | 0.555 | **-0.386** | **0.003** |
| I have spasticity, muscle stiffness | 0.035 | 0.793 | **0.461** | **<0.001** | 0.100 | 0.459 | **-0.601** | **<0.001** |
| I am afraid of falling | -0.197 | 0.141 | **0.284** | **0.032** | 0.260 | 0.051 | **-0.335** | **0.011** |
| I am slower | -0.012 | 0.929 | **0.416** | **0.001** | 0.079 | 0.561 | **-0.266** | **0.046** |
| Comorbidities | | | | | | | | |
| I have other medical conditions | -0.060 | 0.659 | **0.535** | **<0.001** | 0.140 | 0.300 | **-0.312** | **0.018** |
| I am in pain | -0.053 | 0.697 | **0.227** | **0.089** | 0.170 | 0.207 | **-0.271** | **0.042** |

Significant correlations are highlighted in bold. ρ: Spearman rho correlation coefficient; LPA: light physical activity; MVPA: moderate-to-vigorous physical activity.
