# Supplementary material for: Association of Barriers, Fear of Falling and Fatigue with Objectively Measured Physical Activity and Sedentary Behavior in Chronic Stroke
Source: J Clin Med. 2021 Mar 23;10(6):1320. doi: 10.3390/jcm10061320 (PMC8005010; doi:10.3390/jcm10061320)
Supplement: Supplementary file 1 [file jcm-10-01320-s001.zip › Suplementary material 3- proof.docx]

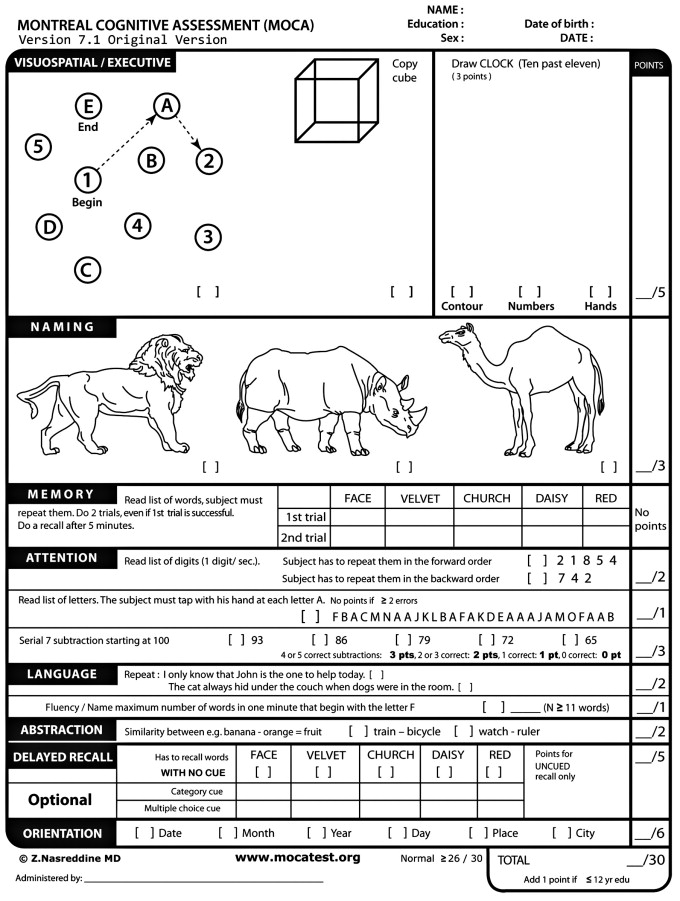


**Figure S1.** Montreal Cognitive Assessment Test (MoCA)

**Table S1.** Charlson Comorbidity Index.

|  | **Score** |  | **Score** |
| --- | --- | --- | --- |
| Myocardial infarction | 1 | Hemiplegia | 2 |
| Congestive heart failure | 1 | Moderate to severe chronic kidney disease | 2 |
| Peripheral vascular disease | 1 | Diabetes -end organ damage | 2 |
| Cerebrovascular accident | 1 | Solid tumor | 2 |
| Dementia | 1 | Leukemia | 2 |
| Chronic obstructive pulmonary disease | 1 | Lymphoma | 2 |
| Connective tissue disease | 1 | Moderate to severe liver disease | 3 |
| Peptic ulcer disease | 1 | Solid, metastatic umor | 6 |
| Mild liver disease | 1 | AIDS | 6 |
| Diabetes mellitus | 1 |  |  |

**Table S2.** The Patient Health Questionnaire (PHQ-9)

| **Over the past 2 weeks, how often have you been bothered by any of the following problems?** | **Not all** | **Several days** | **More than half the days** | **Nearly every day** |
| --- | --- | --- | --- | --- |
| 1. Little interest or pleasure in doing things | 0 | 1 | 2 | 3 |
| 1. Feeling down, depressed or hopeless | 0 | 1 | 2 | 3 |
| 1. Trouble falling asleep, staying asleep, or sleeping too much | 0 | 1 | 2 | 3 |
| 1. Feeling tired or having little energy | 0 | 1 | 2 | 3 |
| 1. Poor appetite or overeating | 0 | 1 | 2 | 3 |
| 1. Feeling bad about yourself - or that you’re a failure or have let yourself or your family down | 0 | 1 | 2 | 3 |
| 1. Trouble concentrating on things, such as reading the newspaper or watching television | 0 | 1 | 2 | 3 |
| 1. Moving or speaking so slowly that other people   could have noticed. Or, the opposite, being so fidgety or restless that you have been moving around a lot more than usual | 0 | 1 | 2 | 3 |
| 1. Thoughts that you would be better off dead of   hurting yourself in some way | 0 | 1 | 2 | 3 |
| 1. If you checked off any problems, how difficult have those problems made it for you to Do your work, take care of things at home, or get along with other people?   ❑ Not difficult at all ❑ Somewhat difficult ❑ Very difficult ❑ Extremely difficult | | | | |

**Table S3.** Stroke Impact Scale-16 (SIS-16).

| **In the past 2 weeks, how difficult was it to...** | Not difficult at all | A little difficult | Somewhat difficult | Very  difficult | Could not do at all |
| --- | --- | --- | --- | --- | --- |
| 1. Dress the top part of your body? | 5 | 4 | 3 | 2 | 1 |
| 1. Bathe yourself? | 5 | 4 | 3 | 2 | 1 |
| 1. Get to the toilet on time? | 5 | 4 | 3 | 2 | 1 |
| 1. Control your bladder (not have an accident)? | 5 | 4 | 3 | 2 | 1 |
| 1. Control your bowels (not have an accident)? | 5 | 4 | 3 | 2 | 1 |
| 1. Standing without losing balance? | 5 | 4 | 3 | 2 | 1 |
| 1. Go shopping? | 5 | 4 | 3 | 2 | 1 |
| 1. Do heavy household chores (e.g. vacuum, laundry or yard work)? | 5 | 4 | 3 | 2 | 1 |
| 1. Stay sitting without losing your balance? | 5 | 4 | 3 | 2 | 1 |
| 1. Walk without losing your balance? | 5 | 4 | 3 | 2 | 1 |
| 1. Move from a bed to a chair? | 5 | 4 | 3 | 2 | 1 |
| 1. Walk fast? | 5 | 4 | 3 | 2 | 1 |
| 1. Climb one flight of stairs? | 5 | 4 | 3 | 2 | 1 |
| 1. Walk one block? | 5 | 4 | 3 | 2 | 1 |
| 1. Get in and out of a car? | 5 | 4 | 3 | 2 | 1 |
| 1. Carry heavy objects (e.g. bag of groceries) with your affected hand? | 5 | 4 | 3 | 2 | 1 |

**Table S4.** Short Falls Efficacy Scale (Short FES-I).

Now we would like to ask some questions about how concerned you are about the possibility of falling. Please reply thinking about how you usually do the activity. If you currently don’t do the activity, please answer to show whether you think you would be concerned about falling IF you did the activity. For each of the following activities, please tick the box which is closest to your own opinion to show how concerned you are that you might fall if you did this activity.

| **In the past 2 weeks, how difficult was it to...** | Not at all concerned | Somewhat concerned | Fairly concerned | Very concerned |
| --- | --- | --- | --- | --- |
| Getting dressed or undressed | 1 | 2 | 3 | 4 |
| Taking a bath or shower | 1 | 2 | 3 | 4 |
| Getting in or out of a chair | 1 | 2 | 3 | 4 |
| Going up or down stairs | 1 | 2 | 3 | 4 |
| Reaching for something above your head or on the ground | 1 | 2 | 3 | 4 |
| Walking up or down a slope | 1 | 2 | 3 | 4 |
| Going out to a social event (e.g. religious service, family gathering or club meeting) | 1 | 2 | 3 | 4 |

**Table S5.** Fatigue severity scale (FSS)

Please circle the number between 1 and 7 which you feel best fits the following statements. This refers to your usual way of life within the last week. 1 indicates “strongly disagree” and 7 indicates “strongly agree.”

| Read and circle a number. | *Strongly Strongly*  *disagree agree* | | | | | | |
| --- | --- | --- | --- | --- | --- | --- | --- |
| 1. My motivation is lower when I am fatigued. | 1 | 2 | 3 | 4 | 5 | 6 | 7 |
| 1. Exercise brings on my fatigue. | 1 | 2 | 3 | 4 | 5 | 6 | 7 |
| 1. I am easily fatigued. | 1 | 2 | 3 | 4 | 5 | 6 | 7 |
| 1. Fatigue interferes with my physical functioning | 1 | 2 | 3 | 4 | 5 | 6 | 7 |
| 1. Fatigue causes frequent problems for me. | 1 | 2 | 3 | 4 | 5 | 6 | 7 |
| 1. My fatigue prevents sustained physical functioning. | 1 | 2 | 3 | 4 | 5 | 6 | 7 |
| 1. Fatigue interferes with carrying out certain duties and responsibilities. | 1 | 2 | 3 | 4 | 5 | 6 | 7 |
| 1. Fatigue is among my most disabling symptoms. | 1 | 2 | 3 | 4 | 5 | 6 | 7 |
| 1. Fatigue interferes with my work, family, or social life | 1 | 2 | 3 | 4 | 5 | 6 | 7 |
